# Predictors of survival in patients with recurrent ovarian cancer undergoing secondary cytoreductive surgery based on the pooled analysis of an international collaborative cohort

**DOI:** 10.1038/bjc.2011.328

**Published:** 2011-08-30

**Authors:** R Y Zang, P Harter, D S Chi, J Sehouli, R Jiang, C G Tropé, A Ayhan, G Cormio, Y Xing, K M Wollschlaeger, E I Braicu, C A Rabbitt, H Oksefjell, W J Tian, C Fotopoulou, J Pfisterer, A du Bois, J S Berek

**Affiliations:** 1 Ovarian Cancer Program, Department of Gynecologic Oncology, Fudan University Cancer Hospital, Shanghai, China; 2 Department of Gynecology and Gynecologic Oncology, Kliniken Essen Mitte, Essen, Germany; 3 Gynecology Service, Department of Surgery, Memorial Sloan-Kettering Cancer Center, New York, NY, USA; 4 Department of Gynecology, Charite Medical University of Berlin, Berlin, Germany; 5 Division of Gynecology Oncology, Norwegian Radium Hospital, University Hospital of Oslo, Oslo, Norway; 6 Department of Obstetrics and Gynecology, Baskent University Faculty of Medicine, Ankara, Turkey; 7 Department of Gynecology, Obstetrics and Neonatology, University of Bari, Bari, Italy; 8 Department of Surgical Oncology, The University of Texas MD Anderson Cancer Center, Houston, TX, USA; 9 Department of Gynecology and Obstetrics, University of Magdeburg, Magdeburg, Germany; 10 Department of Gynecology and Obstetrics, Hospital Solingen, Solingen, Germany; 11 Division of Gynecologic Oncology, Department of Obstetrics and Gynecology, Stanford Cancer Center, Stanford University School of Medicine, Stanford, CA, USA

**Keywords:** ovarian cancer, recurrence, surgery, survival, pooled analysis

## Abstract

**Background:**

This study aims to identify prognostic factors and to develop a risk model predicting survival in patients undergoing secondary cytoreductive surgery (SCR) for recurrent epithelial ovarian cancer.

**Methods:**

Individual data of 1100 patients with recurrent ovarian cancer of a progression-free interval at least 6 months who underwent SCR were pooled analysed. A simplified scoring system for each independent prognostic factor was developed according to its coefficient. Internal validation was performed to assess the discrimination of the model.

**Results:**

Complete SCR was strongly associated with the improvement of survival, with a median survival of 57.7 months, when compared with 27.0 months in those with residual disease of 0.1–1 cm and 15.6 months in those with residual disease of >1 cm, respectively (*P*<0.0001). Progression-free interval (⩽23.1 months *vs* >23.1 months, hazard ratio (HR): 1.72; score: 2), ascites at recurrence (present *vs* absent, HR: 1.27; score: 1), extent of recurrence (multiple *vs* localised disease, HR: 1.38; score: 1) as well as residual disease after SCR (R1 *vs* R0, HR: 1.90, score: 2; R2 *vs* R0, HR: 3.0, score: 4) entered into the risk model.

**Conclusion:**

This prognostic model may provide evidence to predict survival benefit from secondary cytoreduction in patients with recurrent ovarian cancer.

Tumour recurrence occurs in almost all patients with epithelial ovarian cancer (EOC) at a median of 15–18 months from diagnosis (du Bois *et al*, 2009; Hennessy *et al*, 2009). In the past two decades, great efforts have been taken to improve the survival time for platinum-sensitive recurrent (PSR) disease. The most active chemotherapy regimens in the treatment of PSR EOC were reported with a median survival of 29 months (Parmar *et al*, 2003). Although a randomised study, MRC OV05/EORTC 55955 trial, in ovarian cancer of early treatment of relapse based on CA125 level alone *vs* delayed treatment based on conventional clinical indicators showed no survival benefit from early treatment, it was argued that this trial did not appropriately consider the role of SCR since patients with PSR EOC were just assigned to receive chemotherapy (Rustin *et al*, 2010).

There was a reported series of surgery for PSR disease with survival of 20–63.2 months in optimal cytoreductive groups (Berek *et al*, 1983; Morris *et al*, 1989; Jänicke *et al*, 1992; Segna *et al*, 1993; Vaccarello *et al*, 1995; Eisenkop *et al*, 2000; Zang *et al*, 2004; Chi *et al*, 2006; Harter *et al*, 2006; Salani *et al*, 2007; Oksefjell *et al*, 2009; Tian *et al*, 2010), which showed a prolonged survival when parallelly compared with the patients received chemotherapy alone after recurrence (Parmar *et al*, 2003; Pfisterer *et al*, 2006; Alberts *et al*, 2008; Power *et al*, 2009). These data supported the inclusion of secondary cytoreductive surgery (SCR) as a considerable and acceptable therapeutic approach for PSR EOC.

However, the role of SCR remains in controversial because selection bias remains in the surgical cohort and there is no level I/II evidence for SCR in the management of PSR EOC. There also existed some limitations in the publications about SCR because the majority of the literature was retrospective; all of the publication were from single institute; SCR was carried out less frequently in patients with recurrent disease; and most of the published series had fewer than 100 patients. The study with the largest sample was reported by the Arbeitsgemeinschaft Gynaekologische Onkologie (AGO) (Harter *et al*, 2006). About 2500 patients with regard to SCR for PSR EOC were reported in the past three decades.

The aims of this study were to redefine the role of SCR and to establish a predictive model for survival based on the pooled data from an international collaborative cohort. We tried to collect as many individual data as possible, and the final data set used for the pooled analysis included nine studies with a total of 1252 cases from seven centres or groups around the world.

## Materials and methods

### Study design and data collection

Study protocol was developed before data collection. Platinum-sensitive recurrent is defined as the first recurrence after completion of primary treatment of at least 6 months without any evidence of disease. Secondary cytoreductive surgery is performed to remove as much of the tumour as possible in order to prolong survival in patients with PSR EOC.

A systematic search of literature was performed to identify all published studies with no restriction on language. This study protocol was started in December 2008, followed by literature searched in PubMed/Medline, Ovid, Web of knowledge and Embase, with additional MESH and free text terms for ‘secondary cytoreductive surgery and ovarian cancer or secondary cytoreductive surgery and ovarian carcinoma’, were supplemented by hand searches of conference proceedings, reference lists in the publications and review articles. We sent the invitation letters to all available investigators or groups of studies we identified, who had reported articles with regard to SCR. An international collaborative study group was then set up. Our collaborators were closely involved with the main international gynaecological cancer researchers through the Gynecologic Cancer Intergroup and were asked whether they knew of additional published or unpublished studies.

Individual patient data were collected from all participating groups in which these involved already complete data sets. The data collected were all factors according to the review of relative literatures, their clinical relevance, as well as the pilot results obtained based on the studies from the lead group. Data provided by each centre should include at least the following variables: age at recurrence, histology type at primary diagnosis, grade at primary diagnosis, FIGO (the International Federation of Gynecology and Obstetrics) stage at primary diagnosis, residual disease at primary cytoreduction (0 *vs* >0 cm), progression-free interval (PFI), ECOG (the Eastern Cooperative Oncology Group) performance before SCR, ascites at recurrence, CA125 at recurrence, extent status of recurrent tumour (categorised as *localised* which defined as ⩽3 lesions at recurrence *vs multiple* defined as >3 lesions), residual disease after SCR, the largest diameter of the maximal recurrent tumour, bowel resection at SCR, blood loss at SCR, length time of SCR operation from ‘knife to skin’, complications of SCR, follow-up status, survival after SCR and survival after primary therapy. The year of the data was defined as the median year of the data in each study. The updated survival data from original reports were collected if they were available. The residual disease after SCR was defined as: R0, complete resection of all visible disease; R1, remaining small volume disease of 0.1–1 cm; R2, remaining disease >1 cm or residual disease could not be evaluated as used in our previous study (Tian *et al*, 2010).

Data that had been reported previously and some additional updates from each centre were collected and merged after the approval by institutional review board at each participating centre. Data of 2002–2006 from Fudan University Cancer Hospital and Nord-Ostdeutsche Gesellschaft für Gynäkologische Onkologie (NOGGO) were recently published and updated (Sehouli *et al*, 2008; Supplementary Table S1). Eligibility was reviewed for all patients collected. The extensive data validity and consistency were checked for every study before combining for the final analysis. Patients were excluded if the histology of the tumour was not epithelial type, PFI <6 months or the survival data were not available.

### Statistical analysis, derivation and validation of the risk model

The primary outcome measure was survival time from the date of SCR to the date of death. Patients alive were censored on the date of the last follow-up. The univariate analysis for all SAS software (version 9.2, SAS Institute Inc., Cary, NC, USA) was used to develop the risk model for survival. The median survival was computed using the Kaplan–Meier method. The differences in survival of 14 variables were assessed by a log-rank test. Estimated survival rates were calculated by Life Table. We also evaluated hazard ratio (HR) using both log-rank method and the Life Table. The Cox regression was conducted to establish the risk model of survival and estimate the HR. CA125 and PFI were treated as continuous variables, and the cut-off points were evaluated by the receiver-operating characteristics (ROC) curve area and the Youden index (Chmura Kraemer, 1992; Schisterman *et al*, 2005).

Multiple imputation has emerged as an appropriate and flexible way of handling missing data. Some researchers avoid imputation approaches because of fears of ‘making up data’. In fact, complete-case analyses require stronger assumptions than does imputation. Multiple imputation is one technique becoming increasingly advocated to deal with missing data because of its improved performance over alternative approaches (Rubin, 1987; Klebanoff and Cole, 2008; Graham, 2009). The sequential regression multiple imputation (SRMI) method was used to estimate missing values, and the analyses were performed using the multiple-imputed data sets (He *et al*, 2010).

In the final multivariate model, potential predictive variables of a *P-*value <0.05 were considered as risk factors, and a risk score was calculated based on its beta-coefficients from the multivariate analysis. The risk model combining all scores was then developed, and a score for every patient represented the sum of scores for each risk factor.

We adopted an internal validation to assess the performance of the risk model for survival. Youden index is a global measure of diagnostic effectiveness, which calculated by sensitivity+specificity−1. This index represents the maximum vertical distance between the ROC curve and the diagonal line. As it occurs at the cut-point that optimises the variable's differentiating ability when equal weight is given to sensitivity and specificity, we used both the Youden index and ROC curves area to determine the cut-off of the continuous variables included in the risk model.

## Results

### Description of study cohort

By April 2009, seven centres from China (the lead group), Germany, the United States, Norway, Italy and Turkey had joined the international collaborative study group. There were 1252 patients initially collected from nine studies. However, 152 patients were excluded, of which 5 patients with were excluded because of the histology of the tumour being malignant mixed mullerian type, 114 patients for PFI <6 months and the other 33 patients for unavailable survival data. As a result, 1100 patients were finally entered into the study cohort, in which 60 (1986–1997), 87 (1998–2001) and 140 (2002–2006) were from Fudan University Cancer Hospital (FDUCH), respectively; 235 (2000–2003) from AGO; 204 (1999–2004) from NOGGO; 148 (1987–2001) from Memorial Sloan-Kettering Cancer Center; 128 (1985–2000) from Norwegian Radium Hospital; 54 (1990–2001) from Hacettepe University; and 44 (1982–1994) from University of Bari (Supplementary Table S1).

The clinicopathologic characteristics of these patients are presented in Table 1. There were no missing data of PFI, but for other variables the percentage of missing data ranged from 0.2% to 37.9%. The median age at disease recurrence was 56 years, ranging from 16 to 84 years. A median PFI was 19 months (range, 6–326 months). Complete SCR was achieved in 433 (39.4%) patients.

### Predictors of survival

At the time of data analysis, 685 (62.3%) patients had died. The median follow-up time was 20.5 months (range, 0.1–278.8 months) (Supplementary Table S1). The median survival after SCR was 28 months.

On the multivariate analysis, four variables PFI, ascites at recurrence, extent of recurrent tumours and residual disease after SCR were finally identified as the predictors for survival (Supplementary Tables S2, S3). The cut-off of PFI determined by ROC was 23.1 months. The median survival after SCR for patients who had PFI of >23.1 months and ⩽23.1 months was 45.0 months and 21.0 months, respectively (HR: 1.72, 95% CI: 1.45–2.03; *P*<0.0001; Supplementary Figure S1). The cut-off of CA 125 level at recurrence was 251.0 U ml^–1^, but it was not a survival determinant by the Cox regression analysis. Patients with localised lesions had a median survival of 43.9 months and the survival in those with multiple lesions found at SCR decreased to 20.0 months (HR: 1.38, 95% CI: 1.16–1.64; *P*<0.0001; Supplementary Figure S2).

**Figure 1:**
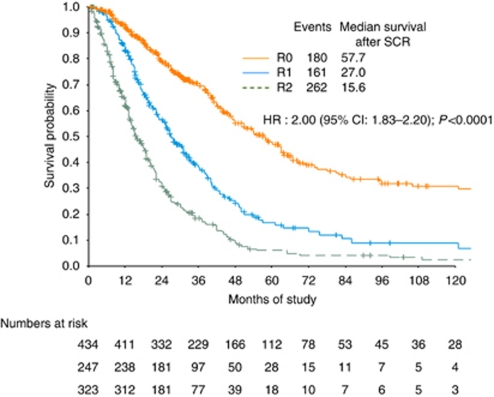
Survival by residual disease after SCR in patients with recurrent ovarian cancer with HR estimated by univariate analysis.

Residual disease after SCR was the strongest survival determinant among almost all those variables, with a median survival of 57.7 months for those without gross residual disease, compared with 27.0 months in R1, and 15.6 months in R2 (R1 *vs* R0: HR: 1.90, 95% CI: 1.54–2.34; *χ*^2^=70.32, *P*<0.0001; R2 *vs* R0: HR: 3.00, 95% CI: 2.43–3.70; *χ*^2^=237.26, *P*<0.0001; R1 *vs* R2: *χ*^2^=36.79, *P*<0.0001; Figure 1).

### The model for survival

Assignment of risk score to each predictor was dividing the coefficient from the Cox regression model by 0.3. Patients with R2 after SCR were given the highest score of 4. Consequently, the total scores for patients with PSR EOC ranged from 0 to 8 points. (Supplementary Table S3)

**Figure 2:**
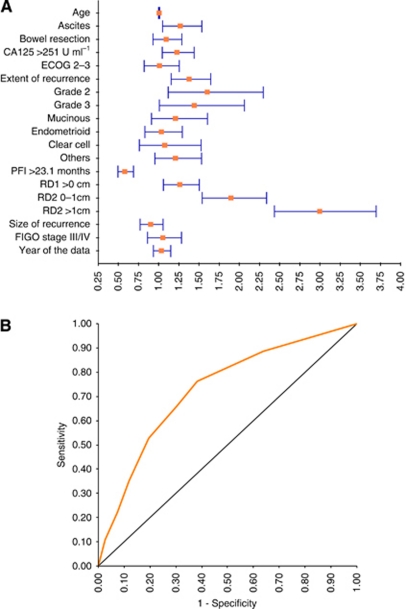
Derivation and validation of the risk model for survival in patients with recurrent ovarian cancer who underwent secondary surgical cytoreduction. (**A**) Risk ratios of all variables. (**B**) ROC curve of validation. The area under the curve is 0.729. The cut-off point of the risk model is 2.5 with the sensitivity, 1-specificity and Youden index (Sen.+Spe.−1) of 76.3%, 38.3% and 0.38, respectively. RD1=residual disease after first surgery; RD2=residual disease after secondary surgery.

**Figure 3:**
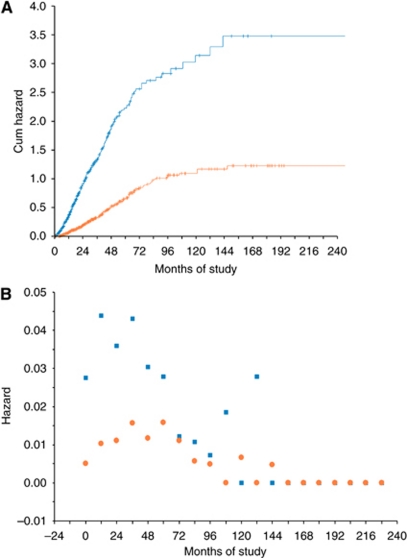
A comparison of survival hazard between low-risk and high-risk groups. (**A**) By log-rank test. (**B**) By the Life Table. Orange line or marker=the curve of patients with scores 0–2 (low risk). Blue line or marker=the curve of patients with scores 3–8 (high risk).

The model performance was internally validated for discrimination. In the risk model, the ROC area was 0.729 (95% CI: 0.698–0.759) with a cut-off point at 2.5 (Table 2, Figure 2). According to the cut-point and the distribution of cases of each score, we divided patients into two classes of low risk (0–2 points) and high risk (3–8 points). In the cohort, there were 418 (38.0%) patients at low risk and 682 patients at high risk. The median survival after SCR for these two classes was 63.0 and 19.1 months, respectively (HR=3.65, *P*<0.0001) (Table 3). Additional analyses the hazard functions of the risk model are further described in Figure 3.

## Discussion

### The risk model and predictors of survival in patients with recurrent ovarian cancer

This risk model suggested residual disease after SCR was the strongest survival determinant when compared with other three factors. It is an evidence to demonstrate to role of secondary cytoreduction. In our pooled analysis, the value of area under the ROC curve suggested that this risk model had a reasonable discriminatory ability and may be used to stratify patients into risk classes for surgical outcomes. Through internal validation, the model showed the feasibility (the ROC curve area was 0.729) in estimating survival determinants for an individual patient with PSR EOC. Using the cut-point scoring system, one patient at low risk with a cut-point value of 0–2 will have a prolonged medial survival of 63 months. To our knowledge, it is the first model of survival for patients with PSR EOC. In this model, PFI, ascites at recurrence, extent of recurrent disease were three intrinsic variables that were well balanced by ethnicities. DESKTOP I found three variables impact on survival after surgery for recurrence: complete resection (residual tumour 0 *vs* >0 mm: HR: 2.94; *P*<0.001), ascites (<500 *vs* >500 ml: HR: 2.30; *P*=0.004) and postoperative chemotherapy (platinum-containing chemotherapy yes *vs* non: HR: 1.84; *P*=0.015). It was different from the results of DESKTOP I that we found extent of recurrent disease was a survival determinant by multivariate analysis (localised lesions *vs* multiple lesions: HR: 1.38, *P*<0.0001). According to the literature, the cut-off value of PFI was generally accepted as 12 months, herein, we demonstrated the cut-off point of PFI being 23.1 months determined by the ROC curve. Of 418 women at low risk, 379 (90.7%) were with R0 after SCR and 39 (9.3%) with R1. That is, no gross residual disease after SCR contributed most to the risk model. Therefore, we believe that SCR should be offered for patients with an estimated complete SCR.

In order to be used easily by clinicians, we tried to establish the model using factors before SCR, but the performance of the model was not satisfactory because of a lower sensitivity and a higher false-positive rate (data not shown). That is, we cannot ignore the variables of the intervention in the derivation of the model. On the multivariate analysis, Figure 2A shows patients with R2 experienced the highest HR of 3.0 (95% CI: 2.43–3.70).

In this cohort, we compared the difference in survival among seven centres, and the *P-*value was <0.0001, but the HR was 0.986 (95% CI: 0.982–0.990) (data was not shown). In our analysis, the year of the data was not a survival determinant, and it was different from that in the meta-analysis by Bristow *et al* (2009). Surgical experience is very important, but this factor has been balanced by patient selection criteria.

There existed some limitations in this study, and we tried to settle those problems before our data analysis. First was about the missing data. It had been acknowledged there would be missing data as not all variables defined in the protocol were collected in all original published studies. It was reasonable to assume the missing data were random and distributed evenly among categories. Compared with simpler approaches such as complete-case analysis, multiple imputation techniques will generally provide more accurate estimates of associations in the data (Rubin, 1987). When we performed the statistical analysis, we used the SRMI method to reduce the impact of missing data.

Second, we did not include salvage chemotherapy before or after SCR as a prognostic factor when gathered the SCR data from each centre. Only a few series reported the systemic postoperative chemotherapy (Jänicke *et al*, 1992; Segna *et al*, 1993; Zang *et al*, 2000; Zang *et al*, 2004; Harter *et al*, 2006; Oksefjell *et al*, 2009). And in most of the publications involved salvage chemotherapy after surgery, there was significant difference in survival when compared with the cycles of salvage chemotherapy, but we considered that those published studies were heterogeneous and the study duration was different. As there are varied regimens for recurrent ovarian cancer, even for one patient, the regimens would change based on the response to chemotherapy. Thus, it is hard to well evaluate the role of salvage chemotherapy in this study cohort. Therefore, we did not consider salvage chemotherapy as a prognostic factor but rather a confounder to have been controlled in the model.

### The role of complete secondary cytoreduction (R0 *vs* R1 and R2)

In this collaborative cohort, survival difference was observed in each stratum among R0, R1 and R2. Surely, a R0 patient most likely is going to live longer after secondary cytoreduction than a R1 or R2 patient. Patients with R1 could also get a significant survival advantage from SCR when compared with those with R2. In 2004, we had reported a prospective study of 117 patients with the same results between R1 and R2, but there was no significant statistical difference in survival between patients with R0 and R1 (Zang *et al*, 2004). So SCR with residual disease ⩽1 cm was defined as optimal cytoreduction in that study. Chi *et al* (2006) set 0.5 cm as a cut-off point and defined residual disease ⩽0.5 cm as optimal result. Data from their centre showed no statistically significant differences in survival between patients with R0 and patients who had residual disease of 0.1–0.5 cm, as well as among patients who had residual disease of 0.6–1.0 cm, 1.1–2.0 cm and >2 cm. So those results suggested 0.5 cm should be the cut-off of optimal SCR. However, in the report of 267 patients by AGO, Harter *et al* (2006) found that just women with R0 after SCR could get a survival benefit. For patients with R0, the median survival was 45.2 months compared with 19.6 months for patients with R1 and 19.7 months for R2. Nevertheless, in a recent report, we found that when we did the paired comparison of survival for patients with R0, R1 and R2, they all showed significant differences, which was coincident with the results of the present pooled analysis (Tian *et al*, 2010).

In the present pooled-analysis, we demonstrated that R0 patients had a contribution of >90% to the group of patients at low risk for survival. So it is important how to find out those will have complete SCR. Our recent publication has presented another model to predict which patients will undergo a successful SCR (Tian *et al*, 2011).

### Conclusions

The role of SCR is first demonstrated by a risk model for survival for patients with recurrent ovarian cancer. Based on the international multi-centre database, this risk model for survival can well predict that which patients would benefit most. R0 should be goal of SCR. However, external validation and randomised trials are needed to test the prognostic model and the role of SCR for patients with PSR ovarian cancer.

## Figures and Tables

**Table 1:** Patient characteristics

	**No. (%) of subjects**			
**Characteristic**	**Total**	**Death**	**Median survival after SCR (months)** a	***P*-value** b
*Age at recurrence, median 56 years (range 16–84)*				
Age				0.4877
<56	543 (49.4)	341 (62.8)	28.0	
⩾56	557 (50.6)	344 (61.8)	29.0	
Year of the data				0.1299
1988–1993	232 (21.1)	177 (76.3)	23.1	
1994–2000	289 (26.3)	206 (71.3)	34.0	
2001–2006	579 (52.6)	302 (52.2)	27.7	
FIGO stage				
Stage I	148 (13.5)	69 (46.6)	53.4	<0.0001
Stage II	164 (14.9)	100 (70.0)	37.0	
Stage III	702 (63.8)	459 (65.4)	25.3	
Stage IV	63 (5.7)	45 (71.4)	25.3	
Unknown	23 (2.1)	12 (52.2)	30.1	
Histology				
Serous	529 (48.1)	375 (70.9)	27.0	0.3237
Mucinous	45 (4.1)	33 (73.3)	20.0	
Endometrioid	126 (11.5)	81 (64.3)	37.0	
Clear cell	46 (4.2)	33 (71.7)	20.0	
Others	118 (10.7)	89 (75.4)	25.3	
Missing	236 (21.5)	74 (31.4)	31.8	
Grade				0.0001
Grade 1	63 (5.7)	32 (50.8)	54.0	
Grade 2	292 (26.5)	221 (75.7)	25.0	
Grade 3	442 (40.2)	317 (71.7)	28.0	
Missing	303 (27.5)	115 (38.0)	31.8	
Primary cytoreduction				<0.0001
0 cm	287 (26.1)	131 (45.6)	42.0	
>0 cm	396 (36.0)	268 (67.7)	22.0	
Missing	417 (37.9)	286 (68.6)	29.0	
PFI				<0.0001
6–23.1 months	647 (58.8)	450 (69.6)	21.0	
>23.1 months	453 (41.2)	235 (51.9)	45.0	
ECOG performance status				<0.0001
0	306 (27.8)	133 (43.5)	38.3	
1	407 (37)	261 (64.1)	27.0	
2	108 (9.8)	80 (74.1)	13.4	
3	2 (0.2)	0 (0)	–	
Missing	277 (25.2)	211 (76.2)	28.9	
CA125 (U ml^–1^)				<0.0001
⩽251	512 (46.5)	262 (51.2)	37.7	
>251	226 (20.5)	154 (68.1)	23.0	
Missing	362 (33.0)	269 (74.3)	26.0	
Ascites				<0.0001
Present	174 (15.8)	137 (78.7)	17.0	
Absent	746 (67.8)	405 (54.3)	33.0	
Missing	180 (16.4)	143 (79.4)	29.4	
Extent of recurrent disease				<0.0001
Localised	517 (47.0)	261 (50.5)	43.9	
Multiple	526 (47.8)	385 (73.2)	20.0	
Missing	57 (5.2)	39 (68.4)	28.0	
Max diameter of the largest recurrent site				0.0680
⩽5 cm	501 (45.5)	279 (55.7)	33.2	
>5 cm	299 (27.2)	179 (59.9)	26.0	
Missing	300 (27.3)	227 (75.7)	24.4	
Bowel resection during SCR				0.7504
None	725 (65.9)	460 (63.4)	28.0	
Yes	373 (33.9)	225 (60.3)	29.6	
Missing	2 (0.2)	0 (0)	–	
Residual disease after SCR				<0.0001
R0	433 (39.4)	179 (41.3)	57.7	
R1	247 (22.4)	161 (65.2)	27.0	
R2	323 (29.4)	262 (81.1)	15.6	
Missing	97 (8.8)	83 (85.6)	17.0	
Total	1100	685 (62.3)	28.0	

Abbreviations: FIGO=International Federation of Gynecology and Obstetrics; ECOG=Eastern Cooperative Oncology Group; PFI=progression-free interval; SCR=secondary cytoreductive surgery; R0=complete resection of all visible disease; R1=remaining small volume disease of 0.1–1cm; R2=remaining disease >1cm.

a The median survival after SCR was computed using Kaplan–Meier method.

b Log-rank test. Missing data were ignored in the analysis.

**Table 2:** Scoring system for survival in patients with recurrent ovarian cancer undergoing secondary cytoreductive surgery

	**Scoring** a			
**Impact factors**	**0**	**1**	**2**	**4**
PFI	>23.1		⩽23.1	
Ascites	Absent	Present		
Extent of recurrent disease	Localised	Multiple		
Residual disease after SCR^b^	R0		R1	R2

Abbreviations: PFI=progression-free interval; SCR=secondary cytoreductive surgery.

a Low-risk: 0–2; high-risk: 3–8.

b R0=complete resection of all visible disease; R1=remaining small volume disease of 0.1–1 cm; R2=remaining disease >1 cm.

**Table 3:** Performance of survival risk model in the validation cohorts

**Scores**	**No. (%)**	**Death (%)**	**Median survival after SCR (months)** a	**HR (95% CI)** b	***P*-value** b
**0∼2**	418 (38.0)	162 (38.8)	63.0	Reference	
**3∼8**	682 (62.0)	523 (76.7)	19.1	3.65 (3.05–4.36)	<0.001
**Total**	1100	685 (62.3)	28.0		–

Abbreviations: CI=confidence interval; HR=hazard ratio; SCR=secondary cytoreductive surgery.

a The median survival after SCR was computed using Kaplan–Meier method.

b The results were computed using Cox Regression analysis.

## References

[bib1] Alberts DS, Liu PY, Wilczynski SP, Clouser MC, Lopez AM, Michelin DP, Lanzotti VJ, Markman M (2008) Randomized trial of pegylated liposomal doxorubicin (PLD) plus carboplatin versus carboplatin in platinum-sensitive (PS) patients with recurrent epithelial ovarian or peritoneal carcinoma after failure of initial platinum-based chemotherapy (Southwest Oncology Group Protocol S0200). Gynecol Oncol 108: 90–94 17949799 10.1016/j.ygyno.2007.08.075 PMC2276605

[bib2] Berek JS, Hacker NF, Lagasse LD, Nieberg RK, Elashoff RM (1983) Survival of patients following secondary cytoreductive surgery in ovarian cancer. Obstet Gynecol 61: 189–193 6823360

[bib3] Bristow RE, Puri I, Chi DS (2009) Cytoreductive surgery for recurrent ovarian cancer: a meta-analysis. Gynecol Oncol 112: 265–274 18937969 10.1016/j.ygyno.2008.08.033

[bib4] Chi DS, McCaughty K, Diaz JP, Chi DS, McCaughty K, Diaz JP, Huh J, Schwabenbauer S, Hummer AJ, Venkatraman ES, Aghajanian C, Sonoda Y, Abu-Rustum NR, Barakat RR (2006) Guidelines and selection criteria for secondary cytoreductive surgery in patients with recurrent, platinum-sensitive epithelial ovarian carcinoma. Cancer 106: 1933–1939 16572412 10.1002/cncr.21845

[bib5] Chmura Kraemer H (1992) Evaluating Medical Tests: Objective and Quantitative Guidelines. SAGE: Newbury Park, CA

[bib6] du Bois A, Reuss A, Pujade-Lauraine E, Harter P, Ray-Coquard I, Pfisterer J (2009) Role of surgical outcome as prognostic factor in advanced epithelial ovarian cancer: a combined exploratory analysis of 3 prospectively randomized phase 3 multicenter trials: by the Arbeitsgemeinschaft Gynaekologische Onkologie Studiengruppe Ovarialkarzinom (AGO-OVAR) and the Groupe d'Investigateurs Nationaux Pour les Etudes des Cancers de l'Ovaire (GINECO). Cancer 115: 1234–1244 19189349 10.1002/cncr.24149

[bib7] Eisenkop SM, Friedman RL, Spirtos NM (2000) The role of secondary cytoreductive surgery in the treatment of patients with recurrent epithelial ovarian carcinoma. Cancer 88: 144–153 10618617 10.1002/(sici)1097-0142(20000101)88:1<144::aid-cncr20>3.3.co;2-o

[bib8] Graham JW (2009) Missing data analysis: making it work in the realworld. Ann Rev Psychol 60: 549–576 18652544 10.1146/annurev.psych.58.110405.085530

[bib9] Harter P, Bois AD, Hahmann M, Harter P, du Bois A, Hahmann M, Hasenburg A, Burges A, Loibl S, Gropp M, Huober J, Fink D, Schröder W, Muenstedt K, Schmalfeldt B, Emons G, Pfisterer J, Wollschlaeger K, Meerpohl HG, Breitbach GP, Tanner B, Sehouli J (2006) Surgery in recurrent ovarian cancer: the arbeitsgemeinschaft gynaekologische onkologie (AGO) DESKTOP OVAR Trial. Ann Surg Onco l 13: 1702–1710 10.1245/s10434-006-9058-0 17009163

[bib10] He Y, Zaslavsky AM, Harrington DP, Catalano P, Landrum MB (2010) Multiple imputationin a large-scale complex survey: a practical guide. Stat Methods Med Res 19: 653–670 19654173 10.1177/0962280208101273 PMC2891890

[bib11] Hennessy BT, Coleman RL, Markman M (2009) Ovarian cancer. Lancet 374: 1371–1382 19793610 10.1016/S0140-6736(09)61338-6

[bib12] Jänicke F, Hölscher M, Kuhn W, von Hugo R, Pache L, Siewert JR, Graeff H (1992) Radical surgical procedure improves survival time in patients with recurrent ovarian cancer. Cancer 70: 2129–2136 1394042 10.1002/1097-0142(19921015)70:8<2129::aid-cncr2820700820>3.0.co;2-u

[bib13] Klebanoff MA, Cole SR (2008) Use of multiple imputation in theepidemiologic literature. Am J Epidemiol 168: 355–357 18591202 10.1093/aje/kwn071 PMC2561989

[bib14] Morris M, Gershenson DM, Wharton JT, Copeland LJ, Edwards CL, Stringer CA (1989) Secondary cytoreductive surgery for recurrent epithelial ovarian cancer. Gynecol Oncol 34: 334–338 2767525 10.1016/0090-8258(89)90168-6

[bib15] Oksefjell H, Sandstad B, Tropé C (2009) The role of secondary cytoreduction in the management of the first relapse in epithelial ovarian cancer. Ann Oncol 20: 286–293 18725390 10.1093/annonc/mdn591

[bib16] Parmar MK, Ledermann JA, Colombo N, du Bois A, Delaloye JF, Kristensen GB, Wheeler S, Swart AM, Qian W, Torri V, Floriani I, Jayson G, Lamont A, Tropé C (2003) Paclitaxel plus platinum-based chemotherapy versus conventional platinum-based chemotherapy in women with relapsed ovarian cancer: the ICON4/AGO-OVAR-2.2 trial. Lancet 361: 2099–2106 12826431 10.1016/s0140-6736(03)13718-x

[bib17] Pfisterer J, Plante M, Vergote I, du Bois A, Hirte H, Lacave AJ, Wagner U, Stähle A, Stuart G, Kimmig R, Olbricht S, Le T, Emerich J, Kuhn W, Bentley J, Jackisch C, Lück HJ, Rochon J, Zimmermann AH, Eisenhauer E (2006) Gemcitabine plus carboplatin compared with carboplatin in patients with platinum-sensitive recurrent ovarian cancer: an intergroup trial of the AGO-OVAR, the NCIC CTG, and the EORTC GCG. J Clin Oncol 24: 4699–4707 16966687 10.1200/JCO.2006.06.0913

[bib18] Power P, Stuart G, Oza A, Provencher D, Bentley JR, Miller Jr WH, Pouliot JF (2009) Efficacy of pegylated liposomal doxorubicin (PLD) plus carboplatin in ovarian cancer patients who recur within six to twelve months: a phase II study. Gynecol Oncol 114: 410–414 19520420 10.1016/j.ygyno.2009.04.037

[bib19] Rubin DB (1987) Multiple Imputation for Nonresponse in Surveys. John Wiley and Sons: New York, NY

[bib20] Rustin GJ, van der Burg ME, Griffin CL, Rustin GJ, van der Burg ME, Griffin CL, Guthrie D, Lamont A, Jayson GC, Kristensen G, Mediola C, Coens C, Qian W, Parmar MK, Swart AM, MRC OV05, EORTC 55955 investigators (2010) Early versus delayed treatment of relapsed ovarian cancer (MRC OV05/EORTC 55955): a randomised trial. Lancet 376: 1155–1163 20888993 10.1016/S0140-6736(10)61268-8

[bib21] Salani R, Santillan A, Zahurak ML, Giuntoli II RL, Gardner GJ, Armstrong DK, Bristow RE (2007) Secondary cytoreductive surgery for localized, recurrent epithelial ovarian cancer: analysis of prognostic factors and survival outcome. Cancer 109: 685–691 17219441 10.1002/cncr.22447

[bib22] Schisterman EF, Perkins NJ, Aiyi L, Bondell H (2005) Optimal cut-point and its corresponding Youden index to discriminate individuals using pooled blood samples. Epidemiology 16: 73–81 15613948 10.1097/01.ede.0000147512.81966.ba

[bib23] Segna RA, Dottino PR, Mandeli JP, Konsker K, Cohen CJ (1993) Secondary cytoreduction for ovarian cancer following cisplatin therapy. J Clin Oncol 11: 434–439 8445417 10.1200/JCO.1993.11.3.434

[bib24] Sehouli J, Stengel D, Oskay-Oezcelik G, Zeimet AG, Sommer H, Klare P, Stauch M, Paulenz A, Camara O, Keil E, Lichtenegger W (2008) Nonplatinum topotecan combinations versus topotecan alone for recurrent ovarian cancer: results of a phase III study of the North-Eastern German Society of Gynecological Oncology Ovarian Cancer Study Group. J Clin Oncol 26: 3176–3182 18591555 10.1200/JCO.2007.15.1258

[bib25] Tian WJ, Chi DS, Sehouli J, Tropé CG, Jiang R, Ayhan A, Cormio G, Xing Y, Breitbach GP, Braicu EI, Rabbitt CA, Oksefjell H, Fotopoulou C, Meerpohl HG, du Bois A, Berek JS, Zang RY, Harter P (2011) A risk model for secondary cytoreductive surgery in recurrent ovarian cancer: an evidence-based proposal for patient selection. Ann Surg Oncol 2011; e-pub ahead of print 6 July 2011; doi:10.1245/s10434-011-1873-2 10.1245/s10434-011-1873-2 21732142

[bib26] Tian WJ, Jiang R, Cheng X, Tang J, Xing Y, Zang RY (2010) Surgery in recurrent epithelial ovarian cancer: benefits on survival for patients with residual disease of 0.1–1 cm after secondary cytoreduction. J Surg Oncol 101: 244–250 20112269 10.1002/jso.21491

[bib27] Vaccarello L, Rubin SC, Vlamis V, Wong G, Jones WB, Lewis JL, Hoskins WJ (1995) Cytoreductive surgery in ovarian carcinoma patients with a documented previously complete surgical response. Gynecol Oncol 57: 61–65 7705701 10.1006/gyno.1995.1099

[bib28] Zang RY, Li ZT, Tang J, Cheng X, Cai SM, Zhang ZY, Teng NN (2004) Secondary cytoreductive surgery for patients with relapsed epithelial ovarian carcinoma: who benefits? Cancer 100: 1152–1161 15022281 10.1002/cncr.20106

[bib29] Zang RY, Zhang ZY, Li ZT, Chen J, Tang MQ, Liu Q, Cai SM (2000) Effect of cytoreductive surgery on survival of patients with recurrent epithelial ovarian cancer. J Surg Oncol 75: 24–30 11025458 10.1002/1096-9098(200009)75:1<24::aid-jso5>3.0.co;2-l

