# Supplementary material for: Predictors of survival in patients with recurrent ovarian cancer undergoing secondary cytoreductive surgery based on the pooled analysis of an international collaborative cohort
Source: Br J Cancer. 2011 Aug 30;105(7):890–6. doi: 10.1038/bjc.2011.328 (PMC3185944; doi:10.1038/bjc.2011.328)
Supplement: Supplementary Appendix [file bjc2011328x1.doc]

**Supplementary Appendix**

**Table S1 Patient cohorts in published studies**

| **Centre** | **Data years** | **Median follow-up time**  **(months, range)** | **Publication year** | **No**. |
| --- | --- | --- | --- | --- |
| FDUCH  Zang *et al.*, 2000  Zang *et al.*, 2004  Tian *et al.*, 2010 | 1986-1997,  1998-2001,  2002-2006 | 26 (15-98)  16 (5-84)  25.1 (5.9-86.8) | 2000  2004  2010 | 287 |
| AGO (Harter *et al.*, 2006) | 2000-2003 | 19 (0.1-48.9) | 2006 | 235 |
| NOGGO (Sehouli *et al.*, 2008) | 1999-2004 | 19 (1-73) | 2008 | 204 |
| MSKCC (Chi *et al.*, 2006) | 1987-2001 | 36.9 (0.2-125.6) | 2006 | 148 |
| Norwegian Radium Hospital (Oksefjell *et al.*, 2009) | 1985-2000 | 26.1 (1.1- 278.8) | 2009 | 128 |
| Hacettepe University (Ayhan *et al.*, 2006) | 1990-2001 | 18.9 (3.5-142.7) | 2006 | 54 |
| University of Bari (Cormio *et al.*, 2006) | 1982-1994 | 41.5 (8-144) | 1998 | 44 |
| **Total** |  | 20.5 |  | **1100** |

Abbreviations: FDUCH, Fudan University Cancer Hospital; AGO, The Arbeitsgemeinschaft Gynaekologische Onkologie; NOGGO, Nord-Ostdeutsche Gesellschaft für Gynäkologische Onkologie; MSKCC, Memorial Sloan-Kettering Cancer Center

**Table S2** Survival for patients with recurrent ovarian cancer undergoing secondary cytoreductive surgery ^a^

|  |  | Cumulative survival | | | |
| --- | --- | --- | --- | --- | --- |
|  |  | 2 yr (%) | 3 yr (%) | 5 yr (%) | 10 yr (%) |
| Median survival (months) | | **30.4** ^a^ | | | |
| *Residual disease after secondary surgery* | | | | | |
|  | R0 | 79.5 | 70.5 | 48.1 | **31.0** |
|  | R1 | 57.1 | 38.8 | 17.1 | 8.9 |
|  | R2 | 32.7 | 19.1 | 6.2 | 2.3 |
| *PFI (months)* | | | | | |
|  | <=23.1 | 45.2 | 32.7 | 17.0 | 9.2 |
|  | >23.1 | 74.4 | 59.7 | 38.3 | 21.8 |
| *Ascites* | | | | | |
|  | Present | 62.1 | 48.1 | 28.6 | 14.8 |
|  | Absent | 38.3 | 22.2 | 9.9 | 7.4 |
| *Extent of recurrent disease* | | | | | |
|  | Localised | 69.3 | 58.5 | 37.6 | 23.4 |
|  | Multiple | 44.2 | 29.9 | 15.4 | 7.6 |
| **Total** |  | **57.2** | **43.8** | **25.9** | **14.6** |

**^a^** By Life Table, with data imputation

**Table S3 Derivation of the scoring system by the Cox regression analysis**

| **Factors** | **β** | **β/0.3** | **Scores** | **Standard error** | ***P* value** |
| --- | --- | --- | --- | --- | --- |
| *PFI* |  |  |  |  |  |
| ≥23.1 months |  |  |  |  | Reference |
| <23.1 months | 0.619 | 2.06 | 2 | 0.082 | <0.001 |
| *Ascites* |  |  |  |  |  |
| Absent |  |  |  |  | Reference |
| Present | 0.273 | 0.91 | 1 | 0.093 | 0.003 |
| *Extent of recurrent disease* |  |  |  |  |  |
| Localised |  |  |  |  | Reference |
| Multiple | 0.326 | 1.09 | 1 | 0.087 | 0.001 |
| *Residual disease after SCR* |  |  |  |  |  |
| R0 |  |  |  |  | Reference |
| R1 | 0.480 | 1.60 | 2 | 0.094 | <0.001 |
| R2 | 1.137 | 3.79 | 4 | 0.104 | <0.001 |

**Table S4** Up-front chemotherapy and salvage chemotherapy

| **Institution/group** | **No. of cases in initial report** | **No. of cases in current study** | **Period** | **Chemotherapy (primary)*** | **Chemotherapy**  **(recurrent)*** |
| --- | --- | --- | --- | --- | --- |
| FDUCH | 60 | 60 | 1986  -1997 | 38 (63.3%) platinum-based IP qw*4.  56 (93.3%) PAC IV* 3-9 cycles | Pre-SCR: 11, post-SCR: 31  paclitaxel; vindesine, etoposide (VE); vindesine, aclacinomycin, mitomycin (VAM) |
|  | 117 | 87 | 1998  -2002 | All: platinum-based IV | Pre-SCR: 46, post-SCR: all  73 (62.4%) cases platinum-based IP *1-7 cycles  79 (67.5%) cases second-line IV >=1cycle: 43 paclitaxel in 43, etoposide in 15, topotecan in 14(12%), gemcitabine in 7(6.0%), ifosfamide in 6 (5.1%), platinum-docetaxel in 5(4.3%), HCPT in 4 (3.4%), NVB in 4 (3.4%), and hexamethylmelamine in 2(1.7%).  1–2 cycles: 16 cases, 3–5 cycles: 52 cases, > 6 cycles: 9 cases. |
|  | 123 | 140 | 2003  -2006 | 121 (98.4%)  Platinum-based IV | 86 (69.9%) cases received salvage chemo after SCR; regimens: 62 platinum and taxol, platinum single or other platinum combinations in 23 patients |
| AGO | 267 | 235 | 2000.1  -2003.12 | 85.8% platinum-based IV | Preoperative: 31.1% cases more than one prior chemotherapy regimens  Post-SCR: 46.8% platinum-based; 42.7% nonplatinum; 10.5% nonchemo |
| Norway | 217 | 128 | 1985  -2000 | NA | Post-SCR: 70 (40%) Platinum single; 32 (18%) Platinum combination; 17 (10%) Paclitaxel monotherapy; 20 (12%) Paclitaxel combination; 34 (20%) others |
| NOGGO | NA | 204 | 1999  -2004 | NA | NA |
| MSKCC | 157 | 148 | 1987  -2001 | All: platinum-based chemo | Post-SCR: 129 (84%) platinum-based; 21 (14%) nonplatinum-based; 3 (2%) unknown |
| Hacettepe | 64 | 54 | 1990  -2001 | All: 6 cycles  54 (84.4%) platinum-based;  7 (10.9%) nonplatinum-based;  3(4.7%) none | Preoperative: 18 (28.1%). 7 (39.0%) platinum -based; 4 (22.2%) topotecan, 1 (5.5%) vepeside, 2 (11.1%) weekly taxol, 4(22.2%) other non -platinum-based.  Post-SCR: 93.7%. 56 (87.4%) chemo; 4 (6.3%) concommitant chemoradiation;  27(45%) platinum-based IV; 10 (16.6%) weekly taxol; 7 (11.7%) topotecan; 4 (6.7%) vepeside, and 12 (20%) other nonplatinum-based IV |
| Italy | 21 | 44 | 1982  -1994 | NA | NA |
| All |  | 1100 |  | Platinum-based: 726/788 (92.1%) | Pre-SCR as neoadjuvant: 158/508 (31.1%)  Post-SCR: 859/1005 (85.5%)  Platinum-based: 541/859 (63.0%)  Nonplatinum-based: 318/859 (37.0%) |

Abbreviations: No, number of cases; chemo, chemotherapy; FDUCH, Fudan University Cancer Hospital; AGO, The Arbeitsgemeinschaft Gynaekologische Onkologie; NOGGO, Nord-Ostdeutsche Gesellschaft für Gynäkologische Onkologie; MSKCC, Memorial Sloan-Kettering Cancer Center; SCR, secondary cytoreductive surgery; IP, intraperitoneal; IV, intravenous.

* the percentages were from the original papers

**Figure S1.** Kaplan-Meier survival curve by progression-free interval

Events Median survival

after SCR

>23.1 months 235 45.0

6-23.1 months 450 21.0

64743222412670452924181515453369262167117815241352420

**Numbers at risk**

6-23.1 months

≥23.1 months

HR 0.49 (95%CI 0.42-0.57); *P* <0.0001

**Figure S2.** Survival by extent of recurrent disease.

Events Median survival

after SCR

Localised 261 43.9

Multiple 385 20.0

HR 2.11 (95%CI 1.80-2.46); *P* <0.0001

| 517 | 494 | 389 | 245 | 164 | 109 | 73 | 55 | 46 | 35 | 28 |
| --- | --- | --- | --- | --- | --- | --- | --- | --- | --- | --- |
| 526 | 505 | 320 | 168 | 99 | 54 | 39 | 22 | 16 | 13 | 11 |

**Numbers at risk**

Localised

Multiple
